# Uncommon etiologies of infectious tonsillitis in adults

**DOI:** 10.1186/1471-2334-13-S1-P32

**Published:** 2013-12-16

**Authors:** Adriana Bold, Garofița Mateescu, Gabriela Iliescu, Amalia Găman, Daniela Cernea

**Affiliations:** 1 University of Medicine and Pharmacy Craiova, Romania; 2 Emergency Clinical County Hospital Craiova, Romania

## Background

Bacteria of the genus *Actinomyces* that colonize the oral cavity rarely cause infection. Fifty-percent of the cases of actinomycosis are in the cervicofacial area, more common in male patients and in infants. In most of cases, the onset of the disease occurs on the teeth or tonsils. Early diagnosis of cervicofacial actinomycosis has been reported in less than 10% of cases. The aim of this report is to emphasize less common etiologies in the diagnosis of adult patients with recurrent tonsillitis.

## Case report

We report the case of a female patient, age 55, known with recurrent acute tonsillitis, hospitalized after two weeks of throat pain, dysphagia, odynophagia, cervical adenopathy, fever and intense asthenia, who remained symptomatic despite of oral cephalexin therapy. The clinical examination revealed hypertrophic palatine tonsils covered by exudates. After tonsillectomy, the diagnosis was carried out through biopsy of the tonsils. Histopathology revealed the infection with *Actinomyces* confirmed by PAS and Giemsa stains, with reactive hyperplasia of the subjacent lymphoid tissue. Specific rovamycin treatment for actinomycosis was administered for 14 days with general improvement of the clinical status.

## Conclusion

Early diagnosis of actinomycosis enables appropriate and prompt treatment, thus preventing the involvement of other important areas.

